# Inter-daily variability in body composition among young men

**DOI:** 10.1186/s40101-015-0070-6

**Published:** 2015-09-22

**Authors:** Petr Kutáč

**Affiliations:** Human Motion Diagnostics Center, University of Ostrava, Varenska 40a, Ostrava 1, 702 00 Czech Republic

**Keywords:** Variability in body composition, Repeated measurement, Typical error of measurement, Working week

## Abstract

**Background:**

The present study analyses changes in body composition over the course of a working week. The purpose of the study is to identify the size of the changes in the observed parameters by means of typical error of measurement (TE) as the initial value for the interpretation of the detected changes in the repeated measurement in diagnostic practice.

**Methods:**

The researched group consisted of 86 males, aged 21.4 ± 1.0 years. All the participants were free of any medical conditions. The measurement of each participant took place over 1 week from Monday till Friday, in the morning hours. Parameters measured: body mass (BM), total body water (TBW), and body fat (BF). The measurement employed two devices using the bioelectric impedance analysis. These devices were the analyzers Tanita BC 418 MA and Nutriguard MS. In order to assess the differences between the average values, the analysis of repeated measurements was used. To assess the material significance, eta squared was used. TE was used to express the size of the changes in the observed parameters.

**Results:**

A statistically significant difference between the average values of the observed parameters was only detected when using the Tanita BC 418 MA analyzer. Based on the post-hoc tests, these differences in the average values were always detected on Monday and Friday. No material significance was proved, however. The highest TE values were also detected in measurements carried out on Monday and Friday. For BM, the value of TE was 0.6 kg, for TBW 1.0–1.1 %, 0.8–0.9 kg, and for BF 1.2–1.6 %, 1.1–1.3 kg depending on the analyzer used.

**Conclusions:**

The results of the present study demonstrate the stability of parameters of body composition throughout a working week, with the provision that standard measuring conditions are fulfilled. For the purpose of diagnostic practice, when interpreting the results of the repeated measurements, it is advisable to take as provable change caused by the observed factors only the ones whose values exceed the value of a weekly TE or the upper limit of the interval of the measurement reliability.

## Background

Analysis of body composition is a common part of diagnosis in many fields of research. It is used in various fields of medicine [1–3], for evaluation of health-oriented skills [4, 5], assessment of impact of changes in a daily regime or energy supply [6, 7], verification of procedures for body mass reduction [8, 9], and/or verification of the effects of dietary supplements [10]. In sports and games, it has also become an integral part of functional diagnosis of athletes [11]. It is used for evaluating the effects of training on changes in individual fractions of body weight [12–16] or for evaluating the changes of these fractions during the racing (league) season [17–19]. For these reasons, the diagnostic practice often uses a repeated measurement which enables detection of the observed changes.

For interpretation of the measured values and detected differences and assessment of the monitored effects, it is of importance to know the possible factors which may have contributed to the resultant changes. Error of measurement has a significant impact. Practically speaking, no device is able to measure with absolute precision. There are consequently no absolutely precise results for any measurement. Errors must therefore always be allowed for and taken into account when interpreting the results. As regards their effects, two types of errors are distinguished: systemic and accidental. Accidental errors are those whose resources are beyond the control of the researcher, they are also called “uncontrollable.” Systemic errors are those which distort the results consistently the same way under the identical conditions of the repeated measurements. These errors include failures in methods, imprecision of gauges or devices, and failures of the observer [20]. As bioelectric impedance analyzers (BIA) are often used in fieldwork, the failure of the observer (operator) can be easily eliminated as a result of the easy operation of these devices. It is consequently necessary to focus on failures in methods and devices. This fact is reflected in a series of studies which deal with this issue. These studies use repeated measurements to calculate the reliability coefficient [21–24] or typical error of measurement (TE) [25, 26]. TE is recommended by the authors for measurements in the fields of bio-medicine which also cover the measurement of body composition. Its indisputable advantage is the possibility to express the size of errors in the units of the monitored parameters.

When identifying the device (method) failure, the repeated measurements are carried out immediately one after another in order to rule out other factors which may disturb the precision of measurement. This is primarily, however, the issue of repeated measurements with longer intervals, which actually prevail in practice. Since every human being is a biological organism which is variable over the course of time and exposed to a number of behavioral variations, it can be assumed that even the parameters of body composition are, to some degree, subject to change. These parameters include first and foremost body mass (BM) and total body water (TBW). Since TBW is measured with the BIA method as the primary parameter and the other parameters are additionally calculated, these changes may even manifest themselves with these secondary parameters. It is evident that the mere awareness of measurement errors in the device, as detected on the basis of immediately repeated measurements, is insufficient. These potential changes should also be taken into account during the interpretation of the measurement results, primarily those which were carried out with a certain time delay. This would prevent misinterpretations due to confusion of common inter-daily changes with the changes induced by the monitored factors. A series of studies completely miss the determination of these inter-daily changes or at least any reflection thereof.

The purpose of the study is to analyze the variability and determine the size of changes in the parameters of body composition over the course of a week.

## Materials and methods

### Participants

The researched group consisted of 86 males (21.4 ± 1.0 years). Their average body height was 181.1 ± 5.5 cm, body weight 74.7 ± 7.8 kg, and BMI 22.7 ± 1.7 kg/m^2^. The participants were students from various fields of study focused on teaching. They did not participate in any sports activity during the research, not even within their studies. None complained of any medical conditions, took any medicines, or took any dietary supplements. They participated in the research voluntarily and were informed in advance of its procedure. All the participants granted written informed consent to participation in the research. The research was approved by the Ethics Committee at the University of Ostrava and is in compliance with the Declaration of Helsinki.

### Procedures

The participants in the measurements were informed in advance of the conditions that have to be observed prior to the measurement (no alcohol less than 24 h before the measurement, no vigorous exercise less than 12 h prior to the measurement, no food or drink less than 3 h prior to the measurement, urination immediately before the measurement). All the measurements took place in the morning hours (7:30 a.m.–9.00 a.m.). The participants were always measured before breakfast. Every participant was measured from Monday till Friday of 1 week, always at the same time. Body composition parameters measured in the research: body mass (BM), total body water (TBW), and body fat (BF). The measurement employed two BIA analyzers using different frequencies for measurement. These devices were the tetrapolar scale Tanita BC 418 MA (Tanita Corporation, Japan) and Nutriguard MS (DataInpud, Germany). Tanita BC 418 MA is a monofrequency BIA analyzer, which uses the frequency of 50 kHz for measurement and eight electrodes integrated in the handles and the stepping platform for transmission of electric current into the body. Nutriguard MS is a multi-frequency analyzer, using 100 kHz for measurement. To transmit electric current into the body, the device uses four adhesive electrodes. Two electrodes are fixed on the upper limb (wrist), and the other two on the lower limb (foot). As the Nutriguard MS analyzer does not feature any weighing device, the body weight—as the initial parameter—had to be measured on Tanita BC 418 MA. Both the analyzers comply with the applicable European standards (93/42EEC, 90/384EEC) for use in the medical industry. The body height was measured by the anthropometer A-226 (Trystom, Czech Republic). A standardized procedure was used for the determination of the body height [27].

### Statistical processing

Remote examinations were identified by means of a boxplot; the division normality was assessed with the Shapiro-Wilk test. As the normality of data division was not disrupted, the assessment of the differences between the average values was based on an analysis of dispersion of repeated measurements (ANOVA). To assess material significance, eta squared was used (*η*^2^). To assess the correlation of the resultant values of the observed parameters between the individual days, the intraclass correlation (ICC) was used as the correlation of identical variables [28]. To express the size of changes in the observed parameters over the course of the week, the typical error of measurement (TE) [25] was used. TE value was calculated on the basis of the square root of the scalar product of square roots of typical errors (TE^2^) between the pairs of trials and degrees of freedom (*Df* = 85) divided by the total sum of the degrees of freedom.

Resulting TE: $$ \frac{\sqrt{{\displaystyle \sum_iT{E_i}^2\cdot D{f}_i}}}{{\displaystyle \sum_iD{f}_i}} $$

Evaluation of Eta squared [29]:

0.01 minor difference

0.06 medium difference

0.14 major difference

A materially significant difference was identified as the one equal to *η*^2^ ≥ 0.06.

The level of statistical significance for all the tests was established as *α* = 0.05. The statistical processing of the results was carried out by means of the program “SPSS Statistic 21.0” (IBM, USA).

## Results

The average values of the observed parameters of the body composition, measured for the individual days, are provided in Table 1. Statistically significant differences for the average values of BF and TBW were detected with the Tanita analyzer (*p* < 0.05). Based on the post-hoc tests, all the cases of these differences in the average values were detected on Monday and Friday. Material significance was not demonstrated in any of the cases; the value *η*^2^ did not even reach the slightest difference (0.01). For the Nutriguard analyzer, the statistical significance between the differences of the average values measured on the individual days was not proved; therefore no material significance was verified.

**Table 1:** Average values of body composition parameters—weekly analysis

Parameters	1. M ± SD	2. M ± SD	3. M ± SD	4. M ± SD	5. M ± SD	*η* ^2^
	Tanita BC 418 MA					
BM (kg)	74.7 ± 7.9	74.6 ± 7.8	74.7 ± 7.7	74.7 ± 7.8	74.7 ± 7.7	–
BF (kg)*	8.4 ± 3.1	8.2 ± 3.0	8.2 ± 2.8	8.2 ± 2.9	7.8 ± 2.8	0.004
TBW (kg)*	48.5 ± 4.4	48.6 ± 4.3	48.7 ± 4.2	48.6 ± 4.4	49.0 ± 4.4	0.001
BF (%)*	11.0 ± 3.4	10.8 ± 3.3	10.8 ± 3.0	10.8 ± 3.1	10.3 ± 3.1	0.005
TBW (%)*	65.1 ± 2.5	65.3 ± 2.4	65.3 ± 2.2	65.3 ± 2.3	65.6 ± 2.3	0.005
	Nutriguard MS					
BF (kg)	12.0 ± 3.1	12.0 ± 3.1	11.9 ± 3.0	12.0 ± 3.0	12.1 ± 2.9	–
TBW (kg)	45.5 ± 3.9	45.4 ± 3.7	45.5 ± 3.6	45.4 ± 3.8	45.3 ± 3.9	–
BF (%)	16.1 ± 3.2	16.1 ± 3.0	16.0 ± 2.8	16.1 ± 2.9	16.2 ± 2.9	–
TBW (%)	60.9 ± 2.3	60.9 ± 2.2	60.9 ± 2.0	60.8 ± 2.1	60.6 ± 2.1	–

*BM* body mass, *BF* body fat, *TBW* total body water, *M* mean, *SD* standard deviation, *η* ^*2*^ eta squared, *1.* Monday, *2.* Tuesday, *3.* Wednesday, *4.* Thursday, *5.* Friday

*ANOVA *p* < 0.05

To analyze the size of changes in the parameters of body composition throughout the week, there was a need to identify the error of measurement of the analyzers employed. To identify the error, the researchers used three immediately repeated measurements on the first day of examination (Monday). To express its size, the calculated average value of TE of these three measurements was used [25]. TE values are provided in Table 2.

**Table 2:** Reliability of measuring the observed body composition parameters

Parameters	Tanita BC 418 MA		Nutriguard MS	
	TE	95 % CI	TE	95 % CI
BM (kg)	0.06	0.05–0.07		
BF (%)	0.36	0.31–0.42	0.15	0.13–0.18
BF (kg)	0.27	0.23–0.31	0.11	0.10–0.13
TBW (%)	0.26	0.23–0.31	0.11	0.10–0.13
TBW (kg)	0.19	0.16–0.22	0.09	0.07–0.10

*BM* body mass, *BF* body fat, *TBW* total body water, *TE* typical error of measurement, *95 % CI* confidence interval

Tables 3 and 4 present the changes in body composition throughout the working week. These are always changes between two consecutive days in a week. The differences between the average values are negligible; they all range within the tolerance level of measurement error. This is also evidenced by the high ICC values. The ICC values explain the 83–100 % dispersion at all parameters (Tables 3 and 4), which is an extremely high narrowness of results [30]. The actual size of the changes in the observed parameters is represented by the TE value.

**Table 3:** Inter-daily changes in parameters of body composition—Tanita BC 418 MA

		Trial				
Parameters		2–1	3–2	4–3	5–4	M
	Diff	−0.1 ± 0.7	0.1 ± 0.6	0.0 ± 0.5	0.0 ± 0.7	
BM (kg)	TE (95 % CI)	0.5 (0.4, 0.6)	0.4 (0.4, 0.5)	0.4 (0.3, 0.5)	0.5 (0.4, 0.6)	0.4 (0.4, 0.5)
	ICC	1.00	1.00	1.00	1.00	1.00
	Diff	−0.2 ± 1.0	0.0 ± 1.1	0.0 ± 0.9	−0.4 ± 0.9	
BF (kg)	TE (95 % CI)	0.7 (0.6, 0.8)	0.8 (0.6, 0.9)	0.7 (0.6, 0.8)	0.6 (0.5, 0.8)	0.7 (0.6, 0.8)
	ICC	0.95	0.94	0.95	0.95	0.95
	Diff	0.1 ± 0.9	0.1 ± 0.9	−0.1 ± 0.8	0.4 ± 0.9	
TBW (kg)	TE (95 % CI)	0.7 (0.6, 0.8)	0.7 (0.6, 0.80)	0.6 (0.5, 0.7)	0.6 (0.5, 0.7)	0.6 (0.6, 0.7)
	ICC	0.98	0.98	0.98	0.98	0.98
	Diff	−0.3 ± 1.3	0.0 ± 1.4	0.0 ± 1.2	−0.5 ± 1.2	
BF (%)	TE (95 % CI)	0.9 (0.8, 1.1)	1.0 (0.8, 1.2)	0.9 (0.8, 1.1)	0.8 (0.7, 1.0)	0.9 (0.8, 1.0)
	ICC	0.93	0.91	0.92	0.93	0.92
	Diff	0.2 ± 0.9	0.0 ± 1.0	0.0 ± 0.9	0.3 ± 0.9	
TBW (%)	TE (95 % CI)	0.7 (0.6, 0.8)	0.7 (0.6, 0.9)	0.7 (0.6, 0.8)	0.6 (0.5, 0.7)	0.7 (0.6, 0.7)
	ICC	0.93	0.91	0.92	0.93	0.92

*BM* body mass, *BF* body fat, *TBW* total body water, *Diff* difference, *TE* typical error of measurement, *95 % CI* confidence interval typical error of measurement, *ICC* intraclass correlation, *M* mean, *1.* Monday, *2.* Tuesday, *3.* Wednesday, *4.* Thursday, *5.* Friday

**Table 4:** Inter-daily changes in parameters of body composition—Nutriguard MS

		Trial				
Parameters		2–1	3–2	4–3	5–4	M
	Diff	0.0 ± 1.1	−0.1 ± 1.0	0.1 ± 1.0	0.1 ± 1.3	
BF (kg)	TE (95 % CI)	0.8 (0.7, 0.9)	0.7 (0.6, 0.9)	0.7 (0.6, 0.9)	1.1 (0.9, 1.3)	0.8 (0.8, 0.9)
	ICC	0.94	0.95	0.95	0.91	0.93
	Diff	−0.1 ± 1.0	0.1 ± 0.8	−0.1 ± 0.9	−0.1 ± 0.9	
TBW (kg)	TE (95 % CI)	0.7 (0.6, 0.90)	0.6 (0.5, 0.7)	0.6 (0.5, 0.8)	0.6 (0.5, 0.8)	0.6 (0.6, 0.7)
	ICC	0.97	0.97	0.97	0.98	0.97
	Diff	0.0 ± 1.5	−0.1 ± 1.4	0.1 ± 1.3	0.1 ± 1.9	
BF (%)	TE (95 % CI)	1.1 (0.9, 1.3)	1.0 (0.8,1.2)	1.0 (0.8, 1.2)	1.1 (1.0, 1.4)	1.0 (0.8, 1.2)
	ICC	0.91	0.92	0.92	0.91	0.92
	Diff	0.0 ± 1.1	0.0 ± 1.0	−0.1 ± 1.0	−0.1 ± 1.4	
TBW (%)	TE (95 % CI)	0.8 (0.7, 1.0)	0.7 (0.6, 0.9)	0.7 (0.6, 0.9)	1.0 (0.8, 1.2)	0.8 (0.7, 0.9)
	ICC	0.91	0.92	0.92	0.91	0.92

*BM* body mass, *BF* body fat, *TBW* total body water, *Diff* difference, *TE* typical error of measurement, *95 % CI* confidence interval typical error of measurement, *ICC* intraclass correlation, *M* mean, *1.* Monday, *2.* Tuesday, *3.* Wednesday, *4.* Thursday, *5.* Friday

## Discussion

Although the present study did not originally focus on the reliability of the employed analyzers, it can be concluded that both the analyzers are sufficiently precise and, as such, suitable for the study. The ICC values at all the observed parameters exceeded 0.9 and thus correspond with the results presented in the series of studies though the authors of those studies carried out the repeated measurements immediately one after another (in a “test-retest” manner) [21, 23, 31–37]. Similar ICC values were detected in the study focused on the correlation of the values measured between 2 days by the Tanita TBF-350 analyzer [38]. The calculated TE values of the immediately repeated measurements are always lower than those given in a series of studies. TE of the BF percentage for the analyzers Tanita BF 350 and BF 410 range from 0.45 to 0.48 % BF; while for Tanita UM-022 the BF value is stated as 0.83 % [35]. TE values measured in the present study with the analyzer. Tanita BC 418 MA corresponds to those presented in the study which focused on the issue and size of TE at the identical type of analyzer [39, 40]; TE values calculated for the analyzers from Nutriguard are even lower. TE values calculated from the measurements between the individual days in a week were higher. This difference is due to an increasing interval between the repeated measurements where the resultant value includes not only the errors of measurement of the analyzer (method) but also the other factors which may primarily include the behavioral variations that are likely to affect the human organism. This is also confirmed by the study which compared the BF proportion measured on 1 day and 2 days. TE value for the repeated measurement on the one day was 0.74 % BF, whereas for the repeated measurement on the second day, it was 1.47 % BF [36]. To detect the actual size of changes in the observed parameters, there would be a need to subtract the TE values, which represent the measurement reliability (Table 2), from the resultant TE values (Tables 3 and 4) between the individual days. This is the method to obtain values which are free of any error of measurement.

**Fig. 1:**
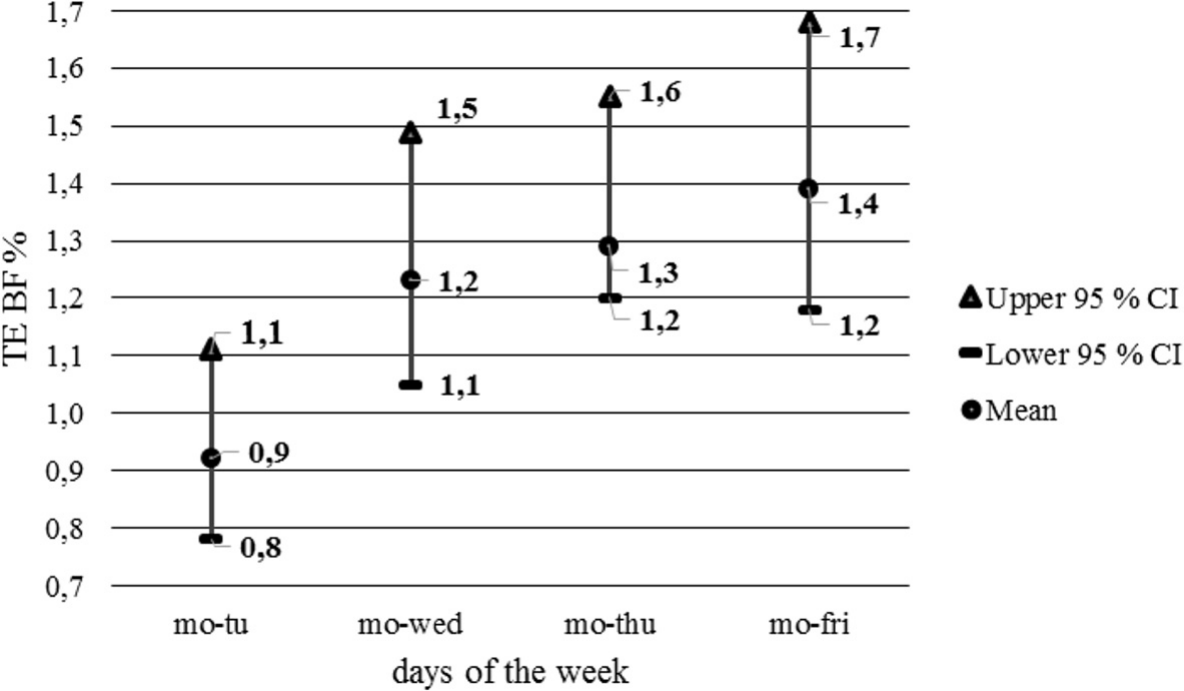
Development of TE BF values, Monday compared with other weekdays—Tanita BC 418 MA: *mo* Monday, *tu* Tuesday, *wed* Wednesday, *thu* Thursday, *fri* Friday, *Upper 95 % CI* upper bound confidence interval, *Lower 95 % CI* lower bound confidence interval, *M* mean, *TE* typical error of measurement, *BF* body fat

**Fig. 2:**
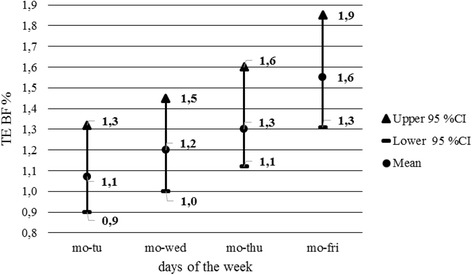
Development of TE BF values, Monday compared with other weekdays—Nutriguard MS: *mo* Monday, *tu* Tuesday, *wed* Wednesday, *thu* Thursday, *fri* Friday, *Upper 95 % CI* upper bound confidence interval, *Lower 95 % CI* lower bound confidence interval, *M* mean, *TE* typical error of measurement, *BF* body fat

The study employed a design that is recommended for calculation of TE by the author of the study [25] and is also defined in the computer program developed by the same author. The program compares the pairs of consecutive trials, i.e., it carries out a comparison of values measured at a 1-day interval. The detected TEs do not provide a fully predictive value for the analysis and assessment of changes in the body composition throughout a week. As stated above and evidenced by the detected values, TE increases with the growing intervals between measurements. This is also demonstrated by the post hoc test results with the Tanita analyzer. To refine the analysis of weekly changes in body composition, the TE calculation was supplemented with a gradual comparison of values measured on Monday with all other days until Friday. TE values increased at all the measured parameters. TE values as calculated from the pairs of measurements on Monday and Friday are provided in Table 5. Development of TE of the BF percentage between the measurements on Monday and on other days is visualized in Figs. 1 and 2.

**Table 5:** Changes in parameters of body composition, Monday to Friday

Parameters	Tanita BC 418 MA		Nutriguard MS	
	TE	95 % CI	TE	95 % CI
BM (kg)	0.6	(0.5, 0.7)		
BF (%)	1.2	(1.1, 1.5)	1.6	(1.3, 1.9)
BF (kg)	1.1	(0.9, 1.3)	1.3	(1.1, 1.5)
TBW (%)	1.0	(0.9,1.2)	1.1	(1.0, 1.4)
TBW (kg)	0.9	(0.8, 1.1)	0.8	(0.7, 1.0)

*BM* body mass, *BF* body fat, *TBW* total body water, *TE* typical error of measurement, *95 % CI* confidence interval

The options for application of the detected TE values in the diagnostic practice can be demonstrated with the results of selected studies which deal with the analysis of the changes in the body composition parameters. The study which focused on the impact of the intervention program on changes in body composition in obese individuals states the statistically significant difference in the BF percentage if this percentage drops by 1.4 % [7]. As demonstrated by the present study results, the drop in the BF percentage by 1.4 % is at the level of TE calculated from the values measured on Monday and Friday and is lower than the upper bound (95 %) of confidence interval of these measurements. From this point of view, the detected difference is negligible. The study in the sphere of sports and games monitors the influence of training on the changes in physical fitness components, with a statistically significant difference stated for a BF percentage drop by 0.9 % [14]. Even in this case, the value of the drop is lower than the calculated TE value, not only for comparison of measurements on Monday and Friday but also for measurements compared among individual days. The presented studies used different analyzers than those employed in the present study, yet both the analyzers applied the BIA method. It can therefore be assumed that TE values should not differ all that much, which is demonstrated by the similarity of the results of the two analyzers, using a different measuring frequency, which were employed in the present study. It has now become evident that the interpretation of results should take account of possible inter-daily changes in the parameters of body composition which can be easily covered by the TE value.

### Limitations of the study

We are aware of the fact that the results of this study may be affected by the number of diagnosed persons, their gender, and the analyzer used. Therefore, the obtained results mainly apply to the analyzer used and the monitored group of the population.

## Conclusion

The results of the present study demonstrate the stability of parameters of body composition throughout a working week, with the provision that the standard measuring conditions are fulfilled. This was also confirmed by the extremely high narrowness of the measurement results between the individual days in a week, as expressed by the ICC values.

For the purpose of diagnostic practice, when interpreting the results of repeated measurements, it is advisable to take as demonstrable change caused by the observed factors only those whose values exceed the value of a weekly TE (measurements from Monday until Friday) and/or the upper bound of the confidence interval of the measurement (95 %). If the difference of the repeated measurements does not exceed the TE level, the detected status shall be evaluated as unchanged.

## Abbreviations

BF: body fat
BIA: bioelectrical impedance analysis
BMI: body mass index
ICC: intraclass correlation
TBW: total body water
TE: typical error of measurement
